# Design of PD‐L1‐Targeted Lipid Nanoparticles to Turn on PTEN for Efficient Cancer Therapy

**DOI:** 10.1002/advs.202309917

**Published:** 2024-03-23

**Authors:** Yelee Kim, Jiwoong Choi, Eun Hye Kim, Wonbeom Park, Hochung Jang, Yeongji Jang, Sung‐Gil Chi, Dae‐Hyuk Kweon, Kyuri Lee, Sun Hwa Kim, Yoosoo Yang

**Affiliations:** ^1^ Biomedical Research Division Korea Institute of Science and Technology (KIST) Seoul 02792 Republic of Korea; ^2^ Department of Life Sciences Korea University Seoul 02841 Republic of Korea; ^3^ Department of Integrative Biotechnology Sungkyunkwan University Suwon 16419 Republic of Korea; ^4^ Division of Bio‐Medical Science and Technology KIST School Korea University of Science and Technology Seoul 02792 Republic of Korea; ^5^ College of Pharmacy and Research Institute of Pharmaceutical Sciences Gyeongsang National University Jinju 52828 Republic of Korea; ^6^ KU‐KIST Graduate School of Converging Science and Technology Korea University Seoul 02841 Republic of Korea

**Keywords:** cancer immunotherapy, lipid nanoparticle, mRNA delivery, tumor‐targeted delivery

## Abstract

Lipid nanoparticles (LNPs) exhibit remarkable mRNA delivery efficiency, yet their majority accumulate in the liver or spleen after injection. Tissue‐specific mRNA delivery can be achieved through modulating LNP properties, such as tuning PEGylation or varying lipid components systematically. In this paper, a streamlined method is used for incorporating tumor‐targeting peptides into the LNPs; the programmed death ligand 1 (PD‐L1) binding peptides are conjugated to PEGylated lipids via a copper‐free click reaction, and directly incorporated into the LNP composition (Pep LNPs). Notably, Pep LNPs display robust interaction with PD‐L1 proteins, which leads to the uptake of LNPs into PD‐L1 overexpressing cancer cells both in vitro and in vivo. To evaluate anticancer immunotherapy mediated by restoring tumor suppressor, mRNA encoding phosphatase and tensin homolog (PTEN) is delivered via Pep LNPs to PTEN‐deficient triple‐negative breast cancers (TNBCs). Pep LNPs loaded with PTEN mRNA specifically promotes autophagy‐mediated immunogenic cell death in 4T1 tumors, resulting in effective anticancer immune responses. This study highlights the potential of tumor‐targeted LNPs for mRNA‐based cancer therapy.

## Graphical abstract

This study investigates a targeted delivery method for cancer therapy, using programmed death ligand 1 (PD‐L1)‐targeting lipid nanoparticles (LNPs) with PD‐L1 binding peptides attached via a copper‐free click reaction. This approach turns on phosphatase and tensin homolog (PTEN) in triple‐negative breast cancers (TNBCs), boosting autophagy, inducing immunogenic cell death (ICD)‐associated damage‐associated molecular patterns (DAMPs), promoting dendritic cell (DC) maturation, and facilitating T‐cell migration to tumors. This findings highlight the promising potential of mRNA‐based cancer therapy using these targeted LNPs.

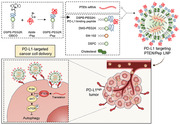

## 1 Introduction

Significant advances in mRNA technology have enabled the rapid and efficient programmed design and manufacturing of functional proteins.^[^ ^1^ ^]^ This versatility has driven the use of mRNA in diverse immunotherapeutic approaches, particularly in the context of cancer immunotherapy.^[^ ^2^ ^]^ mRNA‐based cancer immunotherapy, involving vaccines encoding tumor antigens and immune modulators, has shown promise in both preclinical and clinical studies.^[^ ^3^ ^]^ Furthermore, recent endeavors have emerged to directly address cancer by introducing mRNA‐encoding functional proteins to replace missing or defective endogenous ones.^[^ ^4^ ^]^ For example, delivering mRNAs that encode tumor suppressor genes, such as phosphatase and tensin homolog (PTEN) or p53, to cancer cells with significant deficiencies has proven effective in reducing tumorigenesis by restoring their normal cellular functions.^[^ ^5^ ^]^

To unlock the full therapeutic potential of mRNA, lipid nanoparticles (LNPs) have risen as the most advanced nanocarriers, exemplified by the FDA's approval of COVID‐19 mRNA vaccines.^[^ ^6^ ^]^ Numerous mRNA therapeutics utilizing LNP systems are undergoing clinical trials for a range of human diseases, including cancer.^[^ ^7^ ^]^ Despite the growing utilization of LNPs in mRNA‐based therapies,^[^ ^8^ ^]^ achieving in vivo delivery to specific tissues beyond liver targeting remains a formidable challenge.^[^ ^9^ ^]^

To overcome this issue and effectively transport mRNA to the tumor microenvironment (TME), researchers have devoted significant efforts to engineering the surface of LNPs.^[^ ^9^ ^]^ This modification involves the incorporation of targeting ligands capable of binding to proteins overexpressed on specific cell surfaces.^[^ ^10^ ^]^ In recent years, the phage‐display technique has become a potent method for screening peptides that precisely target certain cells.^[^ ^11^ ^]^ Compared to antibodies, synthetic peptides have several advantages, including lower production costs, increased stability, and better penetration into organs or tumors.^[^ ^12^ ^]^ Here, we selected programmed death ligand 1 (PD‐L1) binding D‐peptides^[^ ^13^ ^]^ to design LNPs targeting cancers overexpressing the PD‐L1 (Pep LNPs). The simplified process for Pep LNPs includes a click reaction‐based conjugation of PEGylated lipids with PD‐L1‐binding peptides, followed by their direct incorporation into the standard LNP preparation procedure. The Pep LNPs were meticulously optimized and evaluated to ascertain whether surface modification could enhance targeted mRNA delivery to PD‐L1 upregulated cancer cells.

To further underscore the therapeutic potential of Pep LNPs, our focus turned to triple‐negative breast cancer (TNBC), a highly aggressive subtype characterized by overexpression of PD‐L1. Molecular profiling has revealed that PTEN is frequently deleted or downregulated in TNBC.^[^ ^14^ ^]^ Elevated PD‐L1 levels are often associated with PTEN loss in many TNBC patients.^[^ ^15^ ^]^ PTEN plays a vital role in inhibiting the oncogenic phosphoinositide 3‐kinase (PI3K)‐protein kinase B (AKT)‐mammalian target of rapamycin (mTOR) signaling pathway, which is involved in abnormal cell growth, metastasis, anti‐autophagy, and an immunosuppressive microenvironment.^[^ ^16^ ^]^ Despite previous efforts to target this pathway through PTEN reactivation in various cancer types,^[^ ^5^, ^17^ ^]^ specific therapeutic effects in TNBC have not been reported.

**Figure 1:**
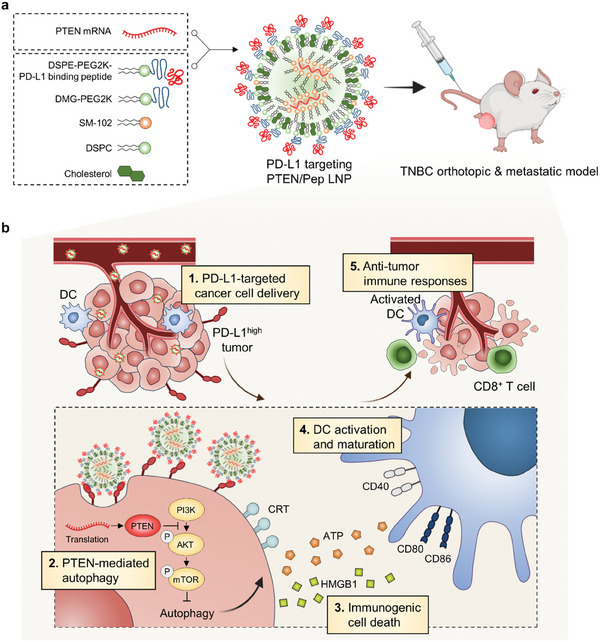
Schematic illustration of PD‐L1‐targeted PTEN/Pep LNPs for immunotherapy of TNBC. a) Scheme for the assembly of Pep LNPs with mRNA. b) Targeted delivery of PTEN/Pep LNPs to PD‐L1‐high tumors induces PTEN‐mediated autophagy and ICD, promotes DC maturation, and stimulates CD8^+^ T cell infiltration, enhancing antitumor immunity.

In this study, we utilized Pep LNPs to specifically deliver PTEN mRNA to a PTEN‐deficient 4T1 TNBC model in vivo. The activation of PTEN in TNBC led to autophagy‐mediated immunogenic cell death (ICD) and elicited robust antitumor immune responses (**Figure** **1**). Given the lack of targeted therapies for TNBC,^[^ ^18^ ^]^ our findings propose Pep LNP‐mediated PTEN mRNA delivery as a tailored option for TNBC treatment. These results emphasize the significance of tumor‐specific delivery by Pep LNPs in maximizing the impact of tumor suppressor mRNA.

## 2 Results

### 2.1 Physicochemical Characterization of Pep LNPs

**Figure 2:**
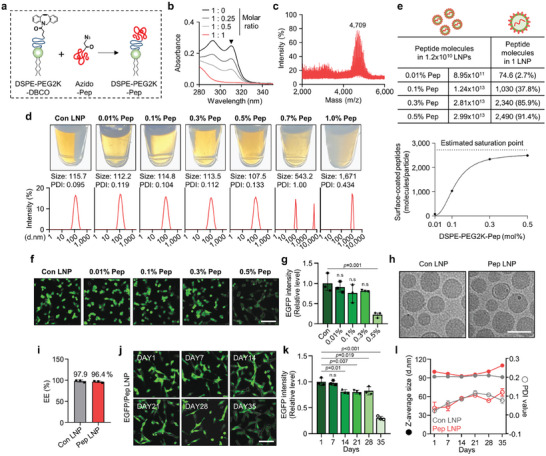
Synthesis condition and characterization of Pep LNPs. a) Schematic illustration showing the synthesis of DSPE‐PEG2K‐Pep. b) UV–vis absorption spectra obtained from click reaction of DSPE‐PEG2K‐DBCO: Azido‐Pep with different molar ratios (1:0, 1:0.25, 1:0.5, 1:1). c) MALDI‐TOFTOF mass spectrometric analysis of DSPE‐PEG2K‐Pep after a 1:1 molar ratio reaction. d) Pictures, size distribution diagram, and average PDI value of Pep LNPs with different DSPE‐PEG2K‐Pep mol% were measured by DLS. e) Quantification of peptides attached to the surface of a single LNP and assessment of coating efficiency through NTA analysis. f) Representative fluorescence images and g) a quantified graph showing the transfection efficiency of EGFP/Pep LNPs in CT26.CL25 cells. Scale bar: 100 µm. h) Representative cryo‐TEM image of Con LNPs and 0.3 mol% Pep LNPs. Scale bar: 100 nm. i) Encapsulation efficiency (EE) of mRNA assessed by RiboGreen assay. j) Representative fluorescence images and k) a quantified graph showing the transfection efficiency of EGFP/Pep LNPs in CT26.CL25 cells at various time points after storage at 4 °C. Scale bar: 100 µm. l) Z‐average size and PDI value of Con LNPs and Pep LNPs measured at different time points after storage at 4 °C. All data presented as mean ± SD, *n* = 3. n.s, not significant; g,k) One‐way ANOVA with Tukey's post‐hoc test.

In this study, we prepared surface‐modified LNPs for selective PD‐L1 targeting. First, to conjugate the PD‐L1 binding peptide with PEGylated lipid, we employed a copper‐free click chemistry technique. This method involved the coupling of DSPE‐PEG2K‐DBCO with the N‐terminal azidoacetyl PD‐L1 binding D‐peptide (NYSKPTDRQYHF; referred to as Pep), resulting in the synthesis of DSPE‐PEG2K‐Pep (**Figure** **2a**). To optimize the reaction conditions, we conducted a comprehensive investigation using various molar ratios (DSPE‐PEG2K‐DBCO: N‐azidoacetyl Pep = 1:0, 1:0.25, 1:0.5, 1:1). The disappearance of the DBCO absorbance peak at 310 nm confirmed the achievement of a highly efficient conjugation reaction at a 1:1 molar ratio (Figure 2b). Additionally, mass spectrometer measurements revealed the transition from the DSPE‐PEG2K‐DBCO peak (3077.83 Da) to the emergence of the DSPE‐PEG2K‐Pep peak (4709.86 Da) after reacting at a 1:1 molar ratio at 37 °C for 1 h (Figure 2c and Figure S1a, Supporting Information). Further validation of the synthesis of DSPE‐PEG2K‐Pep was achieved through ^1^H NMR analysis, which showed the distinct peaks of both hydrocarbons in the lipids (1.2 ppm) and hydroxyl group in serine (9.0 ppm) (Figure S1b, Supporting Information). Thus, we verified a click reaction between azido‐Pep and DSPE‐PEG2K‐DBCO, at a molar ratio of 1:1, is accomplished within an hour.

Next, we employed this DSPE‐PEG2K‐Pep to form PD‐L1‐targeted LNPs (Pep LNPs). The DSPE‐PEG2K‐Pep was added at various ratios (from 0.01 to 1.0 mol% of total lipid) into a mixture of lipid components to prepare Pep LNPs. Using DSPE‐PEG2K‐Pep up to 0.5 mol% concentrations resulted in the production of homogeneous LNPs with little variation in size (107.5–115.7 nm) and a polydispersity index (PDI) value (approximately 0.1) compared to control LNPs (Con LNPs), which are the same LNPs without peptide functionalization. However, LNPs containing DSPE‐PEG2K‐Pep at a concentration above 0.7 mol% showed a noticeable increase in both size and distribution, resulting in visible opacity within the particle solution (Figure 2d). Next, we determined the number of peptide molecules bound to the surface of LNPs by measuring particle number, peptide quantity, and LNP surface area. Initially, we estimated the maximum coverage of peptide molecules on the LNP surface by dividing the surface area of the LNPs by the area of peptides (2724 molecules). Based on this maximum estimate, the average coating efficiency per LNP was also calculated. As shown in Figure 2e, Pep LNPs containing 0.01, 0.1, 0.3, and 0.5 mol% peptides showed surface coating efficiencies of 2.7%, 37.8%, 85.9%, and 91.4%, respectively. By evaluating the transfection efficiency of LNPs containing EGFP mRNA (EGFP/LNPs) in CT26.CL25 cancer cells at various peptide concentrations, a significant decrease in efficiency was noted at 0.5 mol% Pep LNPs (Figure 2f,g). Subsequently, all ensuing experiments were performed using LNPs containing peptides at a concentration of 0.3 mol%, which provided optimal coating saturation.

The resulting Pep LNPs were characterized to evaluate particle morphology, mRNA encapsulation efficiency, and stability. A representative Cryo‐TEM image showed that both Con LNPs and Pep LNPs have a spherical shape with similar sizes (Figure 2h). The encapsulation efficiency of EGFP mRNA in Pep LNPs was assessed by a fluorescent RiboGreen assay, with an average efficiency of 96.4%, a value similar to that of Con LNPs (97.9%) (Figure 2i). For the assessment of particle stability, LNPs were stored at 4 °C for 35 d, during which transfection efficiency, size, and PDI value were monitored. The EGFP protein expression by EGFP/Pep LNP treated CT26.CL25 cells decreased slightly from day 14, persisted until day 28, and then significantly decreased by day 35 (Figure 2j,k). In contrast, the EGFP/Con LNP treated group maintained consistent expression until day 35 (Figure S2a,b, Supporting Information). Concurrently, the size of Pep LNPs displayed a drastic increase after 35 d, whereas Con LNPs maintained a relatively constant size. The PDI value for both LNPs remained below 0.1, indicating a stable particle size distribution (Figure 2l). By confirming that most of the physicochemical parameters of Pep LNPs closely resemble those of Con LNPs for at least one month, we have demonstrated the successful formulation of ligand‐modified LNPs.

### 2.2 Enhanced Cellular Uptake through PD‐L1 Binding Ability of Pep LNPs

**Figure 3:**
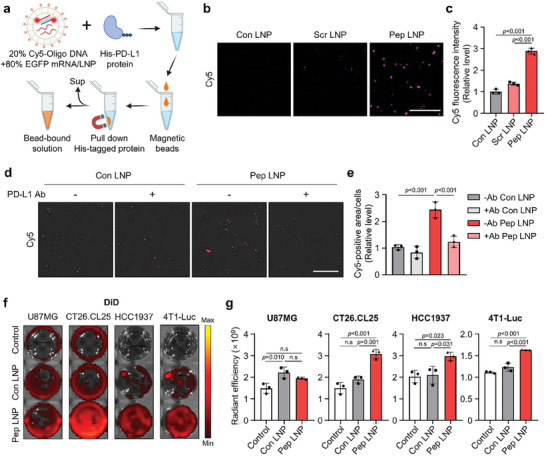
PD‐L1 binding ability of Pep LNPs in vitro. a) Experimental scheme for in vitro PD‐L1 protein binding assay with Cy5‐labeled LNPs. b) Cy5 fluorescence image capturing the bead‐bound solution following incubation of Cy5‐labeled LNPs with His‐PD‐L1 protein. Scale bar: 50 µm. c) The Cy5 fluorescence intensity of bead‐bound solution. d) Cy5 fluorescence image showing the cellular binding of Cy5‐labeled Con or Pep LNPs in CT26.CL25 cells, with or without pre‐treatment of PD‐L1 antibody. Scale bar: 100 µm. e) Quantification of cellular binding assessed by measuring the Cy5‐positive area normalized to the number of cells. f) Well plate images and g) quantification of DiD radiant efficiency in cells treated with DiD‐labeled Luc/Con or Pep LNPs. All data presented as mean ± SD, *n* = 3. n.s, not significant; c,e,g) One‐way ANOVA with Tukey's post‐hoc test.

Next, we assessed whether the peptides bound to the LNP surfaces retained their PD‐L1 binding ability throughout the formulation process, which inevitably involved exposure to ethanol. To investigate this, LNPs containing 20% Cy5‐labeled oligo DNA and 80% EGFP mRNA (Cy5‐labeled LNPs) were incubated with His‐PD‐L1 proteins (**Figure** **3a**). In addition to Con LNPs, LNPs functionalized with a scrambled sequence of PD‐L1 binding peptides (Scr LNPs) were used for comparison. Upon isolating the particles bound to the His‐PD‐L1 proteins using His tag pull‐down magnetic beads, Cy5 signals were observed only in the bead‐bound solution treated with Pep LNPs (Figure 3b,c). The results indicate that the peptides on the LNP surfaces retain PD‐L1 binding ability. Next, we confirmed that Cy5‐labeled Pep LNPs exhibited a significant enhancement in cellular binding to PD‐L1 overexpressing CT26.CL25 cells compared to Con LNPs. This increase was diminished upon pre‐treatment with anti‐PD‐L1 antibodies, which blocked cell surface PD‐L1 proteins (Figure 3d,e, and Figure S3a, Supporting Information). PD‐L1‐dependent cellular binding of Pep LNPs was further validated in both PD‐L1 non‐expressing U87MG and PD‐L1 overexpressing CT26.CL25, HCC1937, and 4T1 cancer cells using DiD‐labeled LNPs (Figure S3a, Supporting Information). While U87MG cells exhibited no difference in DiD fluorescence intensity between Con LNPs and Pep LNPs, a clear increase was detected for Pep LNPs in PD‐L1 overexpressing cancer cells (Figure 3f,g). These results demonstrate that Pep LNPs possess PD‐L1 binding ability, thereby enabling targeted mRNA delivery into the PD‐L1‐expressing cancer cells.

### 2.3 In Vivo Tumor‐Targeted mRNA Delivery of Pep LNPs

After confirming potent PD‐L1 binding ability in vitro, we evaluated the tumor‐targeting effect of Pep LNPs in vivo using both CT26.CL25 colon cancer and 4T1 TNBC‐bearing BALB/c mouse models. First, we verified the stability of Con or peptide‐modified LNPs containing luciferase mRNA (Luc) in 100% mouse serum for 48 h, noting a negligible change in size (Figure S4a, Supporting Information). Given that the peptide‐modified LNPs contain 1.2 mol% of DMG‐PEG2K (C14) and 0.3 mol% DSPE‐PEG2K‐peptide (C18), while Con LNPs solely comprise 1.5 mol% of DMG‐PEG2K, we investigated whether this distinct PEG‐lipid composition influences the biodistribution of LNPs in vivo. As illustrated in Figure S4b,c (Supporting Information), no significant difference in tumor‐localized fluorescent signals was observed between two LNPs with the same composition except for the PEG–lipid composition. Therefore, we confirmed that adding DSPE‐PEG2K does not impact the tumor‐targeting effect of LNPs.

**Figure 4:**
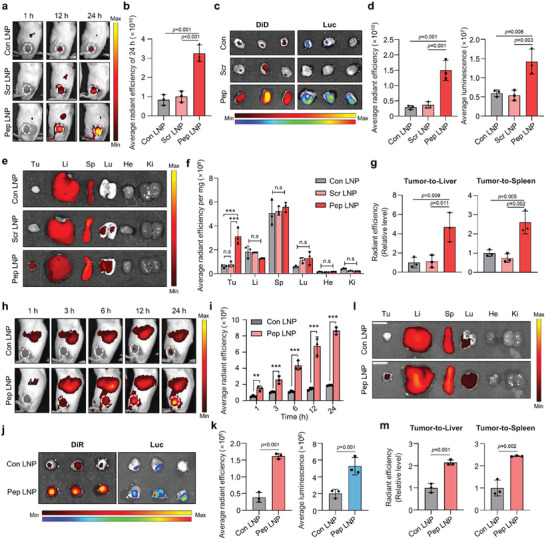
PD‐L1‐high tumor‐targeting of Pep LNPs. a) DiD fluorescence imaging of CT26.CL25 subcutaneous BALB/c mouse model after intravenous injection of DiD‐labeled Luc/Con, Scr, or Pep LNPs (Normalization was performed together for 1 h and 12, 24 h). b) The quantification of tumor‐localized average radiant efficiency of 24 h. c) Fluorescence and luminescence image of tumors harvested at 24 h post‐injection. d) The average radiant efficiency and luminescence of harvested tumors. e) DiD fluorescence imaging of harvested organs and f) quantified graphs of organ DiD radiant efficiency normalized to organ weight (mg). g) Quantified graphs of tumor DiD radiant efficiency normalized to that of the liver or spleen. h) DiR fluorescence imaging and i) quantification of in vivo tumor localized radiant efficiency after intravenous injection of DiR‐labeled Luc/Con or Pep LNPs in BALB/c mice bearing 4T1 tumor. j) DiR fluorescence or luminescence imaging and k) quantification of tumors harvested at 24 h postinjection. l) DiR fluorescence imaging of harvested organs and m) quantification of tumor DiR radiant efficiency with normalized to that of liver or spleen. Li: liver, Sp: spleen, Lu: lung, He: heart. Ki: kidney, Tu: tumor. All data presented as mean ± SD, *n* = 3. n.s, not significant; **p < 0.01, ***p < 0.001. b,d,g) One‐way ANOVA with Tukey's post‐hoc test, f, i) Two‐way ANOVA with Tukey's post‐hoc test. k,m) Unpaired t‐test.

Subsequently, DiD‐labeled Luc/LNPs (Con LNP, Scr LNP, and Pep LNP) were intravenously administered (0.4 mg kg^−1^), and the in vivo distribution in the CT26.CL25 subcutaneous model was monitored. Among the LNPs, Pep LNPs exhibited the highest tumor accumulation 24 h postinjection in vivo (**Figure** **4a,b**). As expected, ex vivo analysis revealed significantly elevated DiD radiant efficiency and luminescence in tumors injected with Pep LNPs compared to those injected with Con LNPs and Scr LNPs (Figure 4c,d). To eliminate the possibility of passive targeting effects due to peptide functionalization, which could potentially prolong circulation time and alter biodistribution,^[^ ^19^ ^]^ we quantified the fluorescence signal of *ex vivo* organs, normalizing it to organ weight (mg). We observed a significant increase in Pep LNP accumulation in tumors without notable variance in other organs (Figure 4e,f). When the ex vivo tumor radiant efficiency was normalized against that of the liver or spleen, considering the high specificity of SM‐102‐based Con LNPs for these organs,^[^ ^20^ ^]^ Pep LNP groups still exhibited a higher tumor‐to‐liver/spleen ratio compared to Con or Scr LNPs (Figure 4g).

Similar results were observed in 4T1 tumor‐bearing mice, with Pep LNPs showing significantly higher tumor accumulation compared to Con LNPs across all time points (Figure 4h,i). The DiR fluorescent signal of Pep LNP‐treated groups in ex vivo tumors after 24 h was 4.18 times higher than that of Con LNPs. Additionally, there was a marked increase in the luminescent signal resulting from Pep LNP accumulation, indicating a 2.64‐fold rise in mRNA translation (Figure 4j,k). The Pep LNP group demonstrated a higher tumor‐to‐liver/spleen ratio in both DiR radiant efficiency and luminescent signal, indicating active tumor‐targeting with Pep LNPs (Figure 4l,m and Figure S4d,e, Supporting Information). The consistently high delivery ratios of tumors to the liver and spleen observed in both tumor models serve as compelling evidence for the apparent tumor‐targeting effect of Pep LNPs. In summary, Pep LNPs exhibited successful accumulation in tumor tissues, primarily driven by PD‐L1‐dependent active targeting mechanisms. This led to efficient mRNA delivery and subsequent translation into target proteins within the tumor sites.

### 2.4 Autophagy Induction in PTEN‐Deficient Cancer Cells by PTEN/Pep LNPs

**Figure 5:**
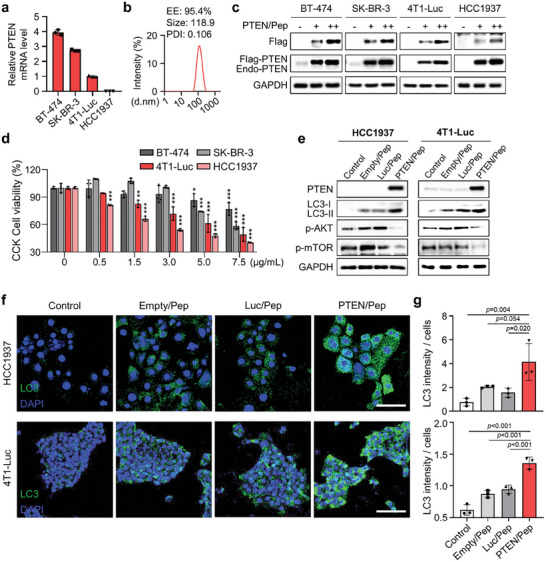
PTEN‐deficient cancer cells experience autophagy via PTEN/Pep LNPs. a) Relative PTEN mRNA expression levels in BT‐474, SK‐BR‐3, 4T1‐Luc, and HCC1937 cells by qPCR. b) The size distribution diagram, average PDI value, and encapsulation efficiency (EE) of PTEN/Pep LNPs. c) Western blot images of cells treated with PTEN/Pep LNPs (0.5, 1 µg mL^−1^). d) CCK cell viability assay for PTEN mRNA‐mediated cell death effect. e) Western blot, f) immunofluorescence images, and g) quantification of LC3 fluorescence intensity per cell numbers of HCC1937 and 4T1‐Luc cells treated with different LNPs. Scale bar: 100 µm. All data presented as mean ± SD, *n* = 3. n.s, not significant; *p < 0.05, **p < 0.01, ***p < 0.001. d,g) One‐way ANOVA with Tukey's post‐hoc test.

Next, we investigated whether the delivery of tumor suppressor PTEN mRNA by Pep LNPs induces anticancer efficacy in four breast cancer cell lines expressing PD‐L1 proteins: PTEN‐null HCC1937, PTEN‐deficient 4T1‐Luc, and PTEN‐competent BT‐474 or SK‐BR‐3 cells (**Figure** **5a** and Figure S3a, Supporting Information). Initially, LNPs containing Flag‐tagged PTEN mRNA (PTEN/Pep LNPs) displayed a diameter of 118.9 nm, PDI value of 0.106, and mRNA encapsulation efficiency of 95.4% (Figure 5b). We then transfected PTEN/Pep LNPs, confirming the successful expression of Flag‐PTEN proteins in BT‐474, SK‐BR‐3, HCC1937, and 4T1‐Luc cells (Figure 5c). Notably, the viability of PTEN‐null HCC1937 and PTEN‐deficient 4T1‐Luc cells significantly decreased upon PTEN mRNA transfection at concentrations of 0.5 and 1.5 µg mL^−1^, respectively. In contrast, the introduction of PTEN mRNA into BT‐474 or SK‐BR‐3 cells did not significantly impact cell viability even at concentrations up to 5 µg mL^−1^ (Figure 5d). These findings support the effectiveness of PTEN restoration in inhibiting the proliferation and survival of cancer cells with defects in PTEN expression while having little impact on PTEN‐competent cells.^[^ ^17d^ ^]^ Next, we investigated whether PTEN restoration induces autophagy by negatively regulating the PI3K‐AKT‐mTOR signaling pathway.^[^ ^21^ ^]^ Treatment of HCC1937 and 4T1‐Luc cells with Pep LNPs for 48 h revealed that only PTEN/Pep LNPs inhibited the phosphorylation of AKT and mTOR. We then assessed the expression of the autophagy marker microtubule‐associated protein light chain 3 (LC3). Significantly, PTEN/Pep LNP treatment elevated LC3 expression compared to both the Empty/Pep LNP and Luc/Pep LNP groups (Figure 5e). Immunofluorescence images also showed a substantial increase in LC3 protein levels in both PTEN/Pep LNP‐treated HCC1937 and 4T1‐Luc cells (Figure 5f,g). These results illustrate that PTEN mRNA treatment in PTEN‐deficient cancer cells promotes autophagy by inhibiting the PI3K‐AKT‐mTOR pathway.

### 2.5 PTEN/Pep LNP Triggered ICD and DC Maturation in TNBC

**Figure 6:**
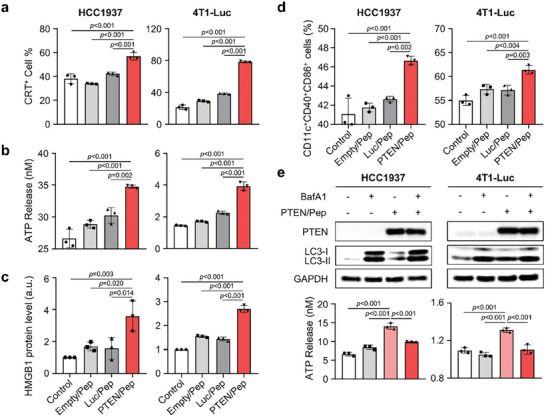
PTEN/Pep LNP‐mediated ICD and DC maturation in TNBC. a–c) Analysis of ICD markers in HCC1937 and 4T1‐Luc cells 24 h (CRT) or 48 h (ATP and HMGB1) after treatment with different LNPs. a) CRT expression on the cell surface was evaluated by flow cytometry. b) Extracellular ATP release was measured by an ATP bioluminescence detection kit. c) Extracellular HMGB1 level was evaluated by western blot analysis. d) BMDCs were added with supernatants from HCC1937 and 4T1‐Luc cells, which had been treated with Empty/Pep LNPs, Luc/Pep LNPs, PTEN/Pep LNPs, or left untreated for 48 h. Then, the percentage of matured DCs (CD11c^+^CD40^+^CD86^+^) was analyzed by flow cytometry. e) HCC1937 and 4T1‐Luc cells were either untreated or treated with PTEN/Pep LNPs for 24 h. Cells were then exposed to BafA1 or left untreated for an additional 12 h before undergoing western blot analysis and extracellular ATP release assay. All data presented as mean ± SD, *n* = 3. a–e) One‐way ANOVA with Tukey's post‐hoc test.

To further investigate whether PTEN could initiate autophagy‐mediated ICD, we evaluated the damage‐associated molecular pattern (DAMP) signals in both cancer cells.^[^ ^22^ ^]^ The results demonstrated that PTEN expression induced by Pep LNPs led to surface calreticulin (CRT) exposure, along with the release of extracellular ATP and high mobility group box 1 (HMGB1) when compared to both the Empty/Pep LNP and Luc/Pep LNP groups (**Figure** **6a–c** and Figure S5a, Supporting Information). In addition, we explored whether PTEN‐induced DAMP release could promote DC maturation. Consistent with the results of DAMP release, only the supernatant from cancer cells treated with PTEN/Pep LNPs significantly enhanced the expression of DC maturation markers, including CD40 and CD86 (Figure 6d). To validate that autophagy induced by PTEN/Pep LNPs was responsible for ICD, we utilized the autophagy inhibitor Bafilomycin A1 (BafA1). BafA1 functions by blocking V‐ATPase, thereby disrupting lysosomal acidification and the autophagy process. This disruption impedes the fusion between autophagosomes and lysosomes, resulting in the accumulation of LC3 on autophagosomal membranes.^[^ ^23^ ^]^ Initially, we confirmed that BafA1 treatment at a concentration of 0.5 × 10^−9^ m effectively inhibited the autophagy process as evidenced by LC3 accumulation, without affecting cell viability (Figure 6e and Figure S5b, Supporting Information). Remarkably, BafA1 treatment reduced the ATP release triggered by PTEN/Pep LNPs. These findings clearly indicate that PTEN/Pep LNPs successfully promote autophagy‐mediated ICD, leading to the generation of DAMP signals that contribute to DC maturation.

### 2.6 Antitumor Efficacy and Immune Responses of PTEN/Pep LNPs in Orthotopic TNBC Model

**Figure 7:**
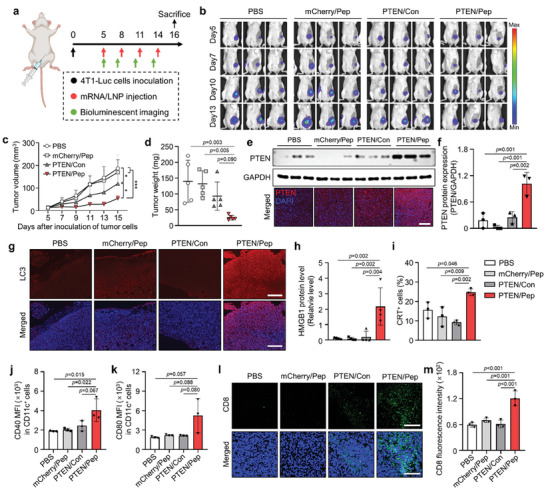
In vivo antitumor immune response of PTEN/Pep LNPs in orthotopic TNBC model. a) Experimental scheme for in vivo study of PTEN/Pep LNPs. 4T1‐Luc tumor‐bearing mice were treated with different LNPs (0.6 mg kg^−1^). b) Bioluminescence imaging of orthotopic 4T1‐Luc tumor‐bearing mice. Imaging was obtained every 3 d from the initial injection day (day 5 after tumor inoculation) until day 13. c) The average tumor growth curve. The size of the tumor was measured on days 5, 7, 9, 11, 13, and 15 post‐inoculation of cancer cells. d) Excised tumor weight. e) Expression of PTEN in tumor tissues, assessed by western blotting and immunofluorescence imaging. Scale bar: 200 µm. f) Quantification of PTEN protein expression levels normalized by the expression of GAPDH. g) Tumor tissues stained with LC3 to evaluate autophagy. Scale bar: 200 µm. h) Relative amounts of HMGB1 in the tumor supernatants were analyzed by western blot and normalized by total protein. i) CRT‐positive cancer cells in tumor tissues were assessed by flow cytometry. j,k) Flow cytometry analysis of CD40 and CD80 expression in CD11c^+^ cells in the TDLN by quantification of median fluorescence intensity (MFI). l) Immunofluorescence imaging of tumor tissues stained with FITC‐conjugated CD8 antibody and m) quantification of CD8 fluorescence intensity. Scale bar: 200 µm. c,d) Data presented as mean ± SD, *n* = 5 mice, h) *n* = 4 mice, f,i–l) *n* = 3. *p < 0.05, ***p < 0.001. c) Two‐way ANOVA with Tukey's post‐hoc test. d,f,h–k,m) One‐way ANOVA with Tukey's post‐hoc test.

Based on the results above, we evaluated the anticancer efficacy and immune responses elicited by PTEN/Pep LNPs in an orthotopic 4T1‐Luc TNBC‐bearing mouse model (**Figure** **7a**). Briefly, the mice were intravenously injected with PBS, mCherry/Pep LNPs, PTEN/Con LNPs, or PTEN/Pep LNPs (0.6 mg kg^−1^) every 3 d for a total of four doses. Simultaneously, the growth of 4T1‐Luc tumors was tracked using bioluminescence imaging. The bioluminescent signal of the tumor region was markedly attenuated in the PTEN/Pep LNPs‐treated group compared to other groups (Figure 7b). Moreover, treatment with PTEN/Pep LNPs led to a 0.47‐fold reduction in tumor volume and a 0.27‐fold decrease in tumor weight compared to the non‐targeted PTEN/Con LNP group (Figure 7c,d and Figure S6a,b, Supporting Information). This effect was further supported by western blot and histological analyses of tumor tissues, revealing the highest PTEN protein expression in the PTEN/Pep LNP‐treated group, thus confirming successful PTEN mRNA delivery via Pep LNPs (Figure 7e,f).

Next, we investigated whether PTEN/Pep LNPs induce autophagy and ICD in tumors. Fluorescence images of tumor tissues stained with LC3 clearly demonstrated elevated autophagy following the PTEN/Pep LNP treatment (Figure 7g). We also confirmed DAMPs from tumor tissues 2 d post‐treatment, which resulted in the increased extracellular release of HMGB1 into the supernatants of the PTEN/Pep LNP‐treated tumors (Figure 7h and Figure S6c, Supporting Information). Additionally, this group exhibited the highest population of CRT‐positive tumor cells than other groups, suggesting the effective induction of ICD by PTEN/Pep LNPs (Figure 7i).

The crucial role of ICD in activating DCs in tumor‐draining lymph nodes (TDLNs) is underscored as a pivotal step in educating tumor‐specific T cells.^[^ ^24^ ^]^ As expected, in the PTEN/Pep LNP‐treated group, there was a significant upregulation in the expression of DC maturation markers, CD40 and CD80, on CD11c^+^ cells within the TDLN, indicating enhanced DC maturation within the TME (Figure 7j,k). Consistent with the aforementioned findings, PTEN/Pep LNP treatment markedly increased the population of infiltrating CD8^+^ T cells throughout the tumor tissues (Figure 7l,m). In conclusion, targeted delivery of PTEN/Pep LNPs efficiently inhibits tumor progression by inducing robust antitumor immune responses, primarily attributable to the effective autophagy‐mediated ICD process.

Lastly, to assess the in vivo safety of PTEN/Pep LNPs, we monitored hematological parameters and conducted a histological assay on major organs, building on the mentioned LNP treatment. Body weight was monitored every 2 d, and no significant changes were observed in any of the treatment groups (Figure S7a, Supporting Information). For histological analysis, major organs (liver, spleen, lung, heart, and kidney) were collected and stained with hematoxylin and eosin (H&E). Our examination revealed no histological differences among the treatment groups, suggesting the absence of notable toxicity (Figure S7b, Supporting Information). Furthermore, the serum hematological tests displayed no obvious changes in any parameters, such as total protein, alanine transaminase (ALT), alkaline phosphatase (ALP), aspartate aminotransferase (AST), blood urea nitrogen (BUN), and creatinine across the groups (Figure S7c, Supporting Information). Collectively, we demonstrated that targeted delivery of PTEN mRNA via Pep LNPs resulted in substantial inhibition of TNBC progression in an orthotopic tumor model with minimal side effects.

### 2.7 Antitumor Effect by PTEN/Pep LNPs in Metastatic TNBC Model

**Figure 8:**
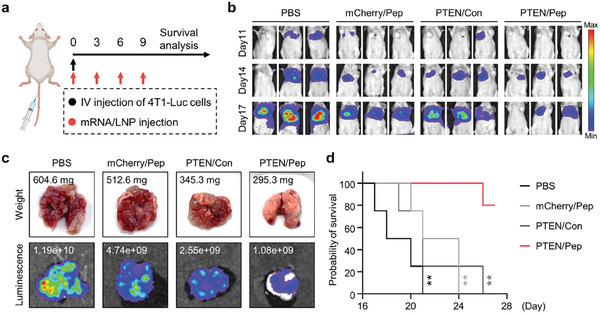
Anti‐metastatic effect of PTEN/Pep LNPs in TNBC lung metastatic model. a) Experimental scheme for in vivo study in a metastatic TNBC model. Mice were injected with 4T1‐Luc cells via the tail vein and treated with different LNPs (0.6 mg kg^−1^). b) Metastatic 4T1‐Luc tumor growth monitored by bioluminescence imaging. c) Ex vivo lung imaging with the bioluminescence flux and weight. d) Mice survival during treatment. Data presented as mean ± SD, *n* = 4 mice; **p < 0.01. d) Log‐rank (Mantel‐Cox) test.

In TNBC, patient mortality rises sharply due to metastatic recurrence, leading to poorer outcomes.^[^ ^25^ ^]^ Additionally, PTEN deficiency promotes epithelial‐mesenchymal transition and metastasis across various cancer types including breast cancer.^[^ ^26^ ^]^ To evaluate the anti‐metastatic potential of PTEN/Pep LNP, mice were injected with 4T1‐Luc cells via tail vein to establish an aggressive metastatic model and treated with different LNP formulations over four doses (**Figure** **8a**). Monitoring the bioluminescent signal in the lung region revealed that PTEN/Pep LNP significantly reduced the lung metastasis compared to the other groups (Figure 8b). On the day 17, when the first mouse in the PBS group has succumbed, mice with similar body weights in the other groups were euthanized for analysis (these were excluded from survival analysis). Remarkably, we observed a substantial reduction in weight and bioluminescent signal in the lung tissue of mice treated with PTEN/Pep LNPs, indicating a decrease in the number of metastatic nodules and inhibition of tumor growth (Figure 8c). Consequently, while mice treated with PBS, mCherry/Pep, and PTEN/Con LNPs exhibited 0% probability of survival on days 21, 24, and 26, respectively, due to respiratory failure from lung cancer, those treated with PTEN/Pep LNPs showed an 80% survival rate until the end of the study (day 27) (Figure 8d). Overall, PTEN/Pep LNP treatment demonstrated effective anti‐metastatic outcomes in a metastatic TNBC model, suggesting therapeutic benefits for the treatment of advanced TNBC patients.

## 3 Discussion

Although PD‐L1/PD‐1 blocking antibodies enhance immune responses by reinvigorating exhausted T cells,^[^ ^27^ ^]^ their efficacy is limited by low tumor immunogenicity and an immunosuppressive TME.^[^ ^28^ ^]^ Current research aims to develop therapeutic modalities inducing ICD to boost antitumor immune responses. Recent studies have emphasized the role of tumor suppressor genes, like PTEN, in antitumor immunity.^[^ ^29^ ^]^ Combining the activation of tumor suppressor genes with immune checkpoint blockade therapy has potentiated the antitumor effects in several studies.^[^ ^5^, ^30^ ^]^ Gene therapy, specifically aimed at restoring tumor suppressor genes' function, uses viral vectors, particularly replication‐deficient adenoviruses.^[^ ^31^ ^]^ These vectors can be administered intratumorally or into body cavities, such as intraperitoneally or intravesically.^[^ ^32^ ^]^ However, challenges include efficient gene transduction within tumors, often hindered by inefficiency and host immune reactions.^[^ ^33^ ^]^

Advancements in LNP systems for mRNA delivery have alleviated concerns about tumor suppressor gene therapy, yet challenges persist in achieving targeted delivery to cancer cells and minimizing off‐target effects. In this study, we developed a PD‐L1‐targeting LNP platform for tumor suppressor gene delivery, integrating peptides with strong PD‐L1 binding affinity^[^ ^13^, ^34^ ^]^ onto the LNP surface by conjugating them with PEGylated lipid, directly incorporating them into the lipid composition. Achieving a high coating efficiency for the ligand is crucial for the effective targeted delivery of lipid‐based nanoparticles.^[^ ^10^, ^34^, ^35^ ^]^ LNPs containing 0.3 mol% DSPE‐PEG2K‐Pep exhibited almost saturated ligand coating efficiency of about 85.9%, ensuring excellent PD‐L1 binding affinity and successful transfection due to the multivalent binding mechanism of the peptides. Incorporating peptides alone enabled SM‐102‐based LNPs, primarily targeted to the liver and spleen, to exhibit high active tumor‐targeting (Figures 2, 3, 4). Exploring the appropriate injection route^[^ ^36^ ^]^ or incorporating lipid components with tumor‐targeting capabilities^[^ ^37^ ^]^ may further enhance selective tumor delivery. Additionally, promoting multivalent binding of an anti‐PD‐L1 antibody or peptide to PD‐L1 on the cell surface can efficiently transport PD‐L1 to lysosomes and induce lysosomal degradation,^[^ ^34^, ^38^ ^]^ offering potential additional immune checkpoint inhibition effects.

In this study, by reactivating PTEN in PTEN‐deficient TNBC, we observed the upregulation of autophagy and ICD‐associated DAMPs, which, in turn, promoted the maturation of DCs and facilitated the migration of T cells toward the tumor site (Figures 5, 6, 7). Moreover, PTEN/Pep LNPs exhibited an anti‐metastatic effect in advanced stage TNBC model, suggesting their potential as a promising therapeutic strategy in TNBC patients (Figure 8). Considering the loss of tumor suppressor gene functions facilitates cancer development,^[^ ^39^ ^]^ the reactivation of these genes offers a distinct advantage. It allows for direct targeting of the root cause associated with underactive or inactive tumor suppression, as opposed to other approaches that target downstream signaling pathways of genes. For instance, the delivery of p53 mRNA significantly delayed tumor growth by inducing cell cycle arrest and apoptosis in p53‐null hepatocellular carcinoma or non‐small cell lung cancer.^[^ ^40^ ^]^

Considering the established clinical correlation between tumor suppressor gene mutations and high‐PD‐L1 expression, such as the link between p53 missense mutation and PD‐L1 expression in TNBC,^[^ ^41^ ^]^ we propose the potential expansion of Pep LNPs’ application in such cases. However, it's crucial to note that subtle changes in the expression of a tumor suppressor gene can impact its function and tumor‐suppressive activity. For example, a 20% decrease in the normal level of PTEN expression is sufficient to cause cancer in the breast, while it may not be low enough to cause carcinogenesis in the liver, small intestine, pancreas, adrenal glands, and prostate. Additionally, since PD‐L1 expression is detected in myeloid cells within TME,^[^ ^42^ ^]^ the selection of the target gene should take into account its potential impact on these cells. For targeted mRNA therapies to appear in the clinic, it will be helpful to find ideal targets based on the patient's tumor genetic profile through tumor genome sequencing. Future work should aim to address challenges related to immunogenicity to enhance the potential of mRNA therapies to progress through clinical trials and meet the demand for tumor suppressor gene upregulation in cancer. Overall, these findings strongly support the promising potential of Pep LNPs as optimal therapeutics for mRNA‐based tumor suppressor gene replacement therapy.

## 4 Experimental Section

### Materials

1,2‐Dimyristoyl‐rac‐glycero‐3‐methoxypolyethylene glycol‐2000 (DMG‐PEG2K, #880151), 1,2‐distearoyl‐sn‐glycero‐3‐phosphoethanolamine‐N‐[dibenzocyclooctyl(polyethylene glycol)−2000] (DSPE‐PEG2K‐DBCO, #880229), and 1,2‐distearoyl‐sn‐glycero‐3‐phosphocholine (DSPC, #850365) were purchased from Avanti Polar Lipids Inc (Alabaster, AL, USA). SM‐102 (#33474) was bought from Cayman Chemical (Ann Arbor, MI, USA). Cholesterol (#C8667) was purchased from Sigma Aldrich (St. Louis, MO, USA). PBS, pH 7.4 (#10010023), DiD (#D7757), and DiR (#D12731) were obtained from Thermo Fishers (Waltham, MA, USA). N‐terminal azidoacetylated PD‐L1 binding D‐peptide (N_3_‐NYSKPTDRQYHF) and its scrambled peptide (N_3_‐RHTNDYSQFYPK) were synthesized by Peptron (Daejeon, Republic of Korea). The pseudouridine (#N‐1019), 5‐methylcytidine (#N‐1014), mCherry mRNA (#L‐7203), EGFP mRNA (#L‐7201), and FLuc mRNA (#L‐7202) modified with 5‐methoxyuridine were purchased from Trilink Biotechnologies (San Diego, CA, USA).

### Synthesis and Characterization of DSPE‐PEG2K‐Pep

DSPE‐PEG2K‐Pep was synthesized using a copper‐free click reaction. DSPE‐PEG2K‐DBCO (5 mM in 50% EtOH, 50 nmol, 1.0 equiv., 10 µL) was mixed with N‐terminal azidoacetylated peptide (10 mg mL^−1^ in RNase‐free water, 50 nmol, 1.0 equiv., 8.18 µL) at 37 °C for 1 h with shaking at 1100 rpm. The resulting mixture was characterized by UV‐vis spectrophotometry (Agilent 8453, Agilent, Stevens Creek Blvd Santan Clara, CA, USA), mass spectrometry (MALDI TOF Voyager DE‐STR, Applied Biosystems, Waltham, MA, USA), and proton nuclear magnetic resonance (Ascend 800 MHz NMR, Bruker magnet system, Billerica, MA, USA).

### LNP Formulation

An ethanol solution with a lipid concentration of 50 × 10^−3^ m was prepared, containing the following components for Con LNPs: SM‐102, cholesterol, DSPC, and DMG‐PEG2K at a molar ratio of 50:38.5:10:1.5, for Pep LNPs: SM‐102, cholesterol, DSPC, DMG‐PEG2K, and DSPE‐PEG2K‐Pep at a molar ratio of 50:38.5:10:1.2:0.3, and for 0.3% DSPE‐PEG2K LNP: SM‐102, cholesterol, DSPC, DMG‐PEG2K, and DSPE‐PEG2K‐DBCO at a molar ratio of 50:38.5:10:1.2:0.3. For the preparation of various mol% compositions of Pep LNPs, the total molar amount of DMG‐PEG2K and DSPE‐PEG2K‐Pep was adjusted to 1.5% mol. For fluorescent labeling, DiR or DiD were added to the ethanol phase at 1 mol% relative to the total lipid. The aqueous phase, containing mRNA, was prepared using a 10 × 10^−3^ m sodium citrate buffer at pH 3. The ionizable lipid: mRNA charge ratio was fixed at 6:1. For the formulation of LNPs without mRNA (Empty/LNPs), the aqueous phase was filled up to the final volume using a 10 × 10^−3^ m sodium citrate buffer at pH 3. The volume ratio between the ethanol phase and aqueous phase was set to 1:2 for NanoAssemblr Spark and 1:3 for NanoAssemblr Ignite formulation device (Precision Nanosystem, Vancouver, Canada). Following this, LNPs were dialyzed twice with 1× PBS (pH 7.4) using a 10 kDa MWCO centrifugal filter for 10 min at 14 000 *g* each time and stored at 4 °C.

### Analysis of Surface‐Bound Peptides and Coating Efficiency

The quantification of surface‐bound peptides on LNPs and their surface coating efficiency were conducted. Initially, various formulations of Pep LNPs were prepared, each containing different percentages (0, 0.01, 0.1, 0.3, 0.5, 0.7, and 1.0 mol%) of DSPE‐PEG2K‐Pep. The size and particle number of these LNPs were determined using a Nanoparticle Tracking Analyzer (NTA, Nanosight NS300, Amesbury, UK). Additionally, the number of peptide molecules in 1.2 × 10^10^ LNPs was measured using the Pierce BCA protein measurement Kit (Pierce Biotechnology, Rockford, IL, USA).

To calculate the number of peptide molecules per single LNP, the peptide quantity was converted into molecules by multiplying with Avogadro's constant (6.022 × 10^23^) and then dividing by the total particle number (1.2 × 10^10^). Given that the hydrodynamic diameter of Pep LNP measured 107.5 nm, the corresponding surface area was calculated by using *S* = 4π*r* ^2^ (36286.63 nm^2^). Considering the estimated hydrodynamic radius of the PD‐L1 binding peptide (1.638 kDa) as 1.03 nm, the corresponding surface area is 13.32 nm^2^. Therefore, it was estimated that achieving 100% coating would allow each LNP to accommodate approximately 2724 peptide molecules on its surface. This estimation served as the basis for calculating the coating efficiency.

### Dynamic Light Scattering

The hydrodynamic diameter and PDI were measured by dynamic light scattering (DLS) using Zetasizer Nano ZS (Malvern Instruments, Malvern, UK). The mRNA/LNPs (2.5 µg of mRNA) were diluted in 1 mL of PBS at pH 7.4 and transferred to a cuvette for size distribution analysis. For stability testing, LNPs were incubated at 4 °C for 35 d, and their stability was monitored via DLS. To assess stability in mouse serum, the same amount of mRNA/LNPs was incubated in 100% mouse serum obtained from BALB/c nude mice and stored at RT.

### Cryogenic Transmission Electron Microscopy (Cryo‐TEM)

The morphology of Pep LNPs was observed using Cryo‐TEM (Tecnai F20 G2, FEI Company, Hillsboro, OR, USA). Pep LNPs were deposited on a thin carbon film covered with copper grids and then vitrified using a Vitrobot (FEI, FP5350). The vitrified samples were kept in liquid nitrogen until the images were captured.

### Encapsulation Efficiency Test

The mRNA encapsulation efficiency was assessed with a Quant‐iT RiboGreen RNA assay Kit (#R11490, Invitrogen, Waltham, MA, USA). Briefly, LNPs were subjected to a 20 min incubation with either 1× TE or 1× TE + 1% Triton X‐100. After the incubation, RiboGreen was added to each sample, and the fluorescence intensity was measured at excitation/emission wavelengths of 485/520 nm using a GloMax plate reader (Promega, Madison, WI, USA). The amount of RNA loaded into the LNPs (internal RNA) was calculated by subtracting the values obtained in 1× TE (external RNA) from 1× TE + 1% Triton X‐100 (total RNA). Subsequently, the encapsulation efficiency was calculated as the percentage obtained by dividing the amount of internal RNA by the total RNA amount.

### Cell Culture and Animals

Mouse CT26.CL25 colorectal carcinoma cell line (#CRL‐2639), 4T1 (#CRL‐2539), 4T1‐Luc (#CRL‐2539‐LUC2) breast cancer cell line, and human U87MG glioblastoma cell line (#HTB‐14) were purchased from American Type Culture Collection ATCC (Manassas, VA, USA). The human HCC1937, BT‐474, and SK‐BR‐3 breast cancer cell lines were obtained from the Korean Cell Line Bank (Seoul, Republic of Korea). All cells were maintained in RPMI‐1640 (Welgene, Gyeongsan, Republic of Korea) supplemented with 10% FBS (Atlas, Fort Collins, TX, USA) and 1% antibiotic‐antimycotic (Gibco, Waltham, MA, USA) and maintained at 37 °C with 5% CO_2_.

BALB/c and BALB/c nude mice were obtained from Orient Bio (Seongnam, Republic of Korea). Mice were fed and housed under pathogen‐free conditions at the Korea Institute of Science and Technology (KIST). All animal experiments were performed and approved following the guidelines of the Institutional Animal Care and Use Committee (IACUC) of the KIST.

### Transfection Efficiency Test

CT26.CL25 cells were seeded in a 6‐well plate and treated with EGFP mRNA/LNPs (1 µg of mRNA) for 24 h. Subsequently, the cells were visualized using an EVOS M5000 fluorescence microscope (Thermo Fishers). To quantitatively analyze the fluorescence images, ImageJ software (National Institutes of Health, NIH, Bethesda, MD, USA) was used.

### PD‐L1 Protein Binding Assay

Cy5‐labeled oligo DNA (5′‐AGCTCTGTTTACGTCCCAGC‐3′) was synthesized by Bioneer (Daejeon, Republic of Korea). Next, an equal amount (300 ng of mRNA) of Con LNPs, Scr LNPs, or Pep LNPs, each containing 20% Cy5‐oligo DNA and 80% EGFP mRNA (based on the mole number of anionic phosphate backbone in nucleic acids), was mixed with recombinant human His‐PD‐L1 protein (2 µg, #ab167713, Abcam, Cambridge, UK) in 1 mL PBS (pH 7.4). This mixture was incubated at 4 °C for 10 min. Subsequently, 25 µL (1 mg) of washed Dynabeads (#10103D, Thermo Fishers) were added to the mixture and incubated at 25 °C for 10 min to capture the His‐tag. The beads were then pulled down by placing the tube on a magnet for 2 min, and the supernatant was discarded. These washing steps were repeated with 1 mL of PBS, and the final beads were resuspended with 100 µL of PBS. Afterward, the Cy5 fluorescence intensity of the bead‐bound solution was measured using GloMax plate readers (Promega), and fluorescence images were captured with an EVOS M5000 microscope (Thermo Fishers).

### Cellular Binding Test

To examine the cellular PD‐L1 binding ability of LNPs, PD‐L1 expressing CT26.CL25 cells were seeded in 6‐well plates and treated with Con or Pep LNPs containing 20% Cy5‐oligo DNA and 80% EGFP mRNA (1 µg of mRNA). As a control group, cells were pretreated with anti‐PD‐L1 antibody (1 mg mL^−1^, #BE0101, BioXCell, Lebanon, NH, USA) for 1 h to block PD‐L1 on the cell membrane. The cellular binding was assessed after 30 min of treatment at 25 °C. Following treatment, the cells were washed twice with PBS and observed using an EVOS M5000 microscope (Thermo Fishers). Fluorescence images were quantitatively analyzed using ImageJ software (NIH).

To further evaluate binding ability, PD‐L1 non‐expressing U87MG and PD‐L1 expressing CT26.CL25, HCC1937, and 4T1‐Luc cells were cultured in a 12‐well plate. The cells were then exposed to Luc mRNA/DiD‐labeled Con or Pep LNPs for 1 h at 37 °C, followed by washing steps with PBS. DiD fluorescence imaging was performed immediately using the IVIS Lumina Series III system (PerkinElmer, Waltham, MA, USA).

### Biodistribution of LNPs

Female BALB/c mice, aged 7 weeks, were subcutaneously inoculated with 1 × 10^6^ 4T1 cells (in 100 µL), while 7 week old male BALB/c mice received subcutaneous inoculation with 2 × 10^6^ CT26.CL25 cells (in 100 µL) in the left flank, initiating tumor growth. When the average tumor volume reached 80 mm^3^, the mice were intravenously injected with DiR or DiD‐labeled Luc mRNA/Con LNPs, Scr LNPs, or Pep LNPs (0.4 mg kg^−1^) or PBS. The biodistribution of LNPs was assessed at specific time points using the IVIS Lumina Series III system. After 24 h postinjection, the mice were sacrificed, and organs and tumors were collected for further analysis. For luminescence imaging of the tumors, luciferin (30 mg mL^−1^, #P1041, Promega) was treated, and imaging was performed simultaneously.

### mRNA Template Construction and In Vitro Transcription

pcDNA3‐Flag‐PTEN (#78777) and pIVT (#122139) plasmids were obtained from Addgene (Watertown, MA, USA). Briefly, the pIVT plasmid underwent site‐directed mutagenesis (SDM) to replace the “GG” initiation sequence of the T7 promoter with “AG,” resulting in the generation of pIVT‐SDM. Subsequently, the PTEN PCR product amplified by primer #1 and #2 (Table S1, Supporting Information) was cloned to the multiple cloning site (cut by BamHI) of the pIVT‐SDM vector using Gibson assembly, resulting in the construction of pIVT‐SDM‐PTEN.^[^ ^43^ ^]^

For in vitro transcription, the template with poly T‐tail (120 nt) was amplified by PCR using primer #3 and #4 (Table S1, Supporting Information). PTEN mRNA was produced using the HiScribe T7 mRNA Kit with CleanCap Reagent AG (#E2080S, NEB, Ipswich, MA, USA), following the manufacturer's protocol. The mRNA's uridine and cytidine were replaced with pseudouridine and 5‐methylcytidine, respectively. The mRNA was purified with Monarch RNA Cleanup Kit (#T2040L, NEB), eluted with RNase‐free DW (1 µg µL^−1^), and stored at −80 °C until use.

### Cytotoxicity Assay

To assess the cytotoxicity of PTEN mRNA, various cancer cells each exhibiting different expression levels of PTEN were seeded in 96‐well plates and transfected with different doses of PTEN mRNA using Lipofectamine MessengerMAX (#LMRNA001, Thermo Fisher). After 24 h of treatment, a solution of Cell Counting Kit‐8 (Dojindo, Tabaru, Japan) was added and quantified at 450 nm with a SpectraMax 34 microplate reader (Molecular Devices, Sunnyvale, CA, USA).

### Validation of Autophagy Using Western Blot and Immunofluorescence

For western blot analysis, HCC1937 and 4T1‐Luc cells were seeded in a six‐well plate at 37 °C. The following day, the culture medium was replaced with a serum‐free medium containing 2.5 µg of Empty/Pep LNPs, Luc/Pep LNPs, PTEN/Pep LNPs, or left untreated, and incubated at 37 °C for 48 h. Subsequently, cells were lysed with 2× SDS, and the cell lysates were separated on SDS‐polyacrylamide gel and then transferred to a nitrocellulose membrane. The western blot analysis employed following antibodies: GAPDH (#MAB5718, R&D, 1:1000), PTEN (#9559S, CST, 1:1000), LC3B (#2775, CST, 1:500), Phospho‐AKT (#9271S, CST, 1:500), Phospho‐mTOR (#2971S, CST, 1:500), Flag (#F1804, Sigma, 1:500), anti‐Rabbit IgG‐HRP antibody (#GTX213110‐01, GeneTex), and anti‐mouse IgG‐HRP antibody (GTX213111‐01, GeneTex).

To visualize the LC3 expression, HCC1937, and 4T1‐Luc cells were plated on 35 mm glass slides for confocal microscopic analysis. The next day, the culture medium was switched to a serum‐free medium including 2.5 µg of Empty/Pep LNPs, Luc/Pep LNPs, PTEN/Pep LNPs, or left untreated, and cells were maintained at 37 °C for 48 h. After the treatment, cells were washed with PBS and fixed for 15 min in 4% PFA solution at RT. Following fixation, the cells were washed with PBS and stained with anti‐LC3B antibody (#2775, CST, 1:200) for 24 h at 4 °C. After staining, the cells were washed with PBS and subsequently stained with Alexa Fluor 488 goat anti‐rabbit IgG (H+L) secondary antibody (#A‐11008, Thermo Fisher) for 1 h at RT, followed by an additional PBS wash. The next step involved another incubation with Hoechst 33 342 (#H3570, Thermo Fisher, 1:1000) for 7 min. Fluorescence imaging was conducted using a Leica TCS SP8 confocal laser scanning microscope (Leica, Germany).

### DAMP Analysis

To determine LNP‐induced cell surface CRT level, HCC1937 and 4T1‐Luc cells were seeded in a six‐well plate and exposed to 2.5 µg of Empty/Pep LNPs, Luc/Pep LNPs, PTEN/Pep LNPs, or left untreated in serum‐deprived media. After 24 h post‐treatment, the cells were washed with PBS and stained with APC‐conjugated CRT antibody (#ab196159, Abcam) at 4 °C for 1 h. Subsequently, cells were washed twice with PBS and analyzed using a CytoFLEX flow cytometer (Beckman Coulter, Brea, CA, USA).

To analyze the release of extracellular HMGB1 and ATP, cells were treated with various LNPs in serum‐free media for 48 h. For the HMGB1 release assessment, the supernatants were collected and subjected to centrifugation at 300 g for 10 min to remove cell debris. Subsequently, the supernatants were concentrated using a 3 kDa MWCO centrifugal filter for 15 min at 4 °C and 14 000 *g*. These concentrated supernatants were then diluted with 5× SDS and subjected to western blot analysis for detection using an anti‐HMGB1 antibody (#ab18256, Abcam). HMGB1 level was normalized by the total protein level detected by Coomassie blue staining (InstantBlue, #ab119211, Abcam). For the investigation of ATP release, the debris‐eliminated supernatants were assessed using the ATP Assay Kit (#FF2000, Promega) following the manufacturer's instructions.

### In Vitro DC Maturation

For the preparation of BMDCs, bone marrow cells were isolated from the hind legs of 7 week old male BALB/c mice using a 3 mL syringe and a 40 µm cell strainer. In addition, RBC lysis buffer (#420301, BioLegend, San Diego, CA, USA) was employed to eliminate cells that were not required. Subsequently, the isolated bone marrow cells were plated in a 100 mm culture dish and allowed to grow overnight in RPMI‐1640 (Welgene) containing 10% FBS (Gibco) and 1% antibiotic–antimycotic (Gibco) (Day 0). Following the isolation of floating bone marrow cells, they were seeded in a 35 mm cell culture dish at a density of 1.2 × 10^6^ cells and treated with GM‐CSF (#315‐03, PeproTech, 20 ng mL^−1^), IL‐4 (#214‐14, PeproTech, 20 ng mL^−1^), and 0.1% β‐mercaptoethanol. On days 3 and 5, half of the medium was replaced with fresh media containing the same formulations. On day 7, the cells were seeded in a 35 mm cell culture dish at a density of 1.0 × 10^6^ cells using the same culture media formulation. Next, BMDCs were added with supernatants from HCC1937 and 4T1‐Luc cells, which had been treated with Empty/Pep LNPs, Luc/Pep LNPs, PTEN/Pep LNPs, or left untreated for 48 h. After 24 h treatment, BMDCs were detached using PBS, and the Fc region of the BMDCs was blocked using mouse BD Fc Block. Subsequently, PE CD11c antibody (#117307, BioLegend), FITC CD40 antibody (#102905, BioLegend), and APC CD86 antibody (#105113, BioLegend) were added and incubated at 4 °C for 1 h. The degree of DC maturation was then assessed using flow cytometry.

### Validation of PTEN‐Mediated Autophagy in ICD with Autophagy Inhibitor

HCC1937 and 4T1‐Luc cells were seeded in a six‐well plate. The next day, the cells were treated with 2.5 µg of Empty/Pep LNPs, Luc/Pep LNPs, and PTEN/Pep LNPs, or left untreated for 24 h. Following the treatment, the culture medium was replaced with a serum‐free medium containing 0.5 × 10^−9^ m of Bafilomycin A1 (#B1793, Sigma) for 12 h. Subsequently, the supernatants underwent an ATP release assay (#FF2000, Promega), while the cells were subjected to western blot analysis.

### In Vivo Antitumor Effect of mRNA/LNPs

To investigate the antitumorigenic effects of various LNPs, 8 week old female BALB/c mice were inoculated with 1 × 10^5^ 4T1‐Luc cells (in 60 µL) into the mammary fat pad. After 5 d, all mice were randomly divided into four groups (*n* = 5). On days 5, 8, 11, and 14 after tumor inoculation, the mice were intravenously injected with either PBS, mCherry/Pep LNPs, PTEN/Con LNPs, or PTEN/Pep LNPs (0.6 mg kg^−1^). Tumor volume and body weights were measured and recorded every other day. Luminescence imaging was conducted on days 5, 7, 10, and 13. For in vivo tumor growth imaging, 100 µL of luciferin (30 mg mL^−1^) was injected intraperitoneally, and imaging was performed 10 min later. On day 16 (2 d after the last injection), the mice were euthanized and the tumors were harvested to examine ICD induction and the number and phenotype of immune cells, such as T cells.

### In Vivo Antitumor Immune Response Analysis

Tumors harvested were transformed into single‐cell suspensions using a tumor dissociation kit (#130‐096‐730, Miltenyi Biotec) and a gentleMACS Octo Dissociator with Heaters (#130‐096‐427, Miltenyi Biotec). To remove RBCs from the suspensions, an RBC lysis buffer (#420301, BioLegend) was applied. TDLNs were harvested and separated into single‐cell suspensions using a plunger. After cell counting, FcBlock was introduced to the cell suspensions to prevent nonspecific binding for 15 min. Subsequently, multiparameter staining was conducted for 1 h at 4 °C using flow cytometry to identify specific populations within the tumor tissues: (i) APC anti‐CRT antibody (#ab196159, Abcam) for CRT^+^ cancer cells and (ii) FITC anti‐CD3 (#100203, BioLegend) and APC anti‐CD8 (#100712, BioLegend) for CD8^+^ T cells, and TDLN: (iii) FITC anti‐CD11c (#117305, BioLegend), APC anti‐CD40 (#124611, BioLegend), or APC anti‐CD80 (#104714, BioLegend) antibodies for matured DCs. The supernatant from tumor homogenates was subjected to western blot analysis to determine the extracellular HMGB1 level, which was subsequently normalized by total protein.

### Immunofluorescence for Tissues

Tumor tissues were isolated from the mice 48 h postinjection to analyze protein expression. A portion of these tissues underwent homogenization followed by subsequent western blot analysis. Simultaneously, another subset of the samples was sliced into 4 µm sections from formalin‐fixed paraffin‐embedded blocks. After antigens were retrieved through boiling, tissue slides were subjected to staining using LC3B‐Alexa Fluor 647 (CST, #18577S, 1:50), PTEN (#9559S, CST, 1:100), or FITC CD8a (#100803, BioLegend) antibodies for 24 h at 4 °C. After the incubation, only the PTEN samples were exposed to Alexa Fluor 647 goat anti‐rabbit IgG (H+L) secondary antibody (#A‐212245, Thermo Fisher) for 1 h at RT. The subsequent step included another incubation with Hoechst 33342 (#H3570, Thermo Fisher, 1:1000) for 7 min. Fluorescence imaging was conducted using an EVOS M5000 fluorescence microscope (Thermo Fishers).

### In Vivo Toxicity Study

In preparation for histological examination, the organs were fixed with 4% PFA and embedded in paraffin. Following this, 4 µm sections were obtained and subjected to H&E staining. The slides were then assessed using an Olympus BX51 microscope (Tokyo, Japan). Regarding the serum biochemical analysis, blood samples were drawn via abdominal vena cava and collected in heparin tubes (#367871, Becton Dickinson). After plasma separation at 3500 *g* for 20 min, the levels of AST, ALT, ALP, BUN, creatinine, and total protein were measured by DKKorea Co. (Anyang, Republic of Korea), a nonclinical contract research organization institution.

### Therapeutic Study in Metastatic Tumor Model

8 week old female BALB/c mice were injected with 5 × 10^4^ 4T1‐Luc cells (in 100 µL) through the tail vein and randomly divided into four groups (*n* = 5) for different treatments. On days 0, 3, 6, and 9 after tumor inoculation, the mice were intravenously injected with either PBS, mCherry/Pep LNPs, PTEN/Con LNPs, or PTEN/Pep LNPs (0.6 mg kg^−1^). Body weights were measured every other day and luminescence imaging was conducted on days 11, 14, and 17. The extracted lung tissues were weighed and subsequently soaked in luciferin (30 mg mL^−1^) for bioluminescence imaging.

### Statistics

The statistical analysis was performed using GraphPad Prism 7.0 software (GraphPad, San Diego, CA, USA). For comparisons between two groups or more, a student's *t*‐test or one‐way analysis of variance (ANOVA) with Tukey's correction was applied, followed by Tukey's post‐hoc test for multiple comparisons. The experimental data are presented as mean ± standard deviation, with each experiment independently repeated at least three times, yielding consistent results. The representative dataset is presented in the results.

## Conflict of Interest

The authors declare no conflict of interest.

## Supporting information

Supporting Information

## Data Availability

The data that support the findings of this study are available in the supplementary material of this article.
