# Supplementary material for: Design of PD‐L1‐Targeted Lipid Nanoparticles to Turn on PTEN for Efficient Cancer Therapy
Source: Adv Sci (Weinh). 2024 Mar 23;11(22):2309917. doi: 10.1002/advs.202309917 (PMC11165541; doi:10.1002/advs.202309917)
Supplement: Supplementary file 1 — Supporting Information [file ADVS-11-2309917-s001.pdf]

ADVANCED SCIENCE
Open Access

# Supporting Information

for *Adv. Sci.*, DOI 10.1002/advs.202309917

Design of PD-L1-Targeted Lipid Nanoparticles to Turn on PTEN for Efficient Cancer Therapy

*Yelee Kim, Jiwoong Choi, Eun Hye Kim, Wonbeom Park, Hochung Jang, Yeongji Jang, Sung-Gil Chi, Dae-Hyuk Kweon, Kyuri Lee, Sun Hwa Kim* and Yoosoo Yang**

# Supporting Information for

# Design of PD-L1-targeted lipid nanoparticles to turn on PTEN for efficient cancer therapy

Yelee Kim[a, b], Jiwoong Choi[a], Eun Hye Kim[a, b], Wonbeom Park[c], Hochung Jang[a, d], Yeongji Jang[a, b], Sung-Gil Chi[b], Dae-Hyuk Kweon[c], Kyuri Lee[e], Sun Hwa Kim[a, f, *] and Yoosoo Yang [a, d, *]

[a]Biomedical Research Division, Korea Institute of Science and Technology (KIST), Seoul 02792, Republic of Korea

[b]Department of Life Sciences, Korea University, Seoul 02841, Republic of Korea

[c]Department of Integrative Biotechnology, Sungkyunkwan University, Suwon 16419, Republic of Korea

[d]Division of Bio-Medical Science and Technology, KIST School, Korea University of Science and Technology, Seoul 02792, Republic of Korea

[e]College of Pharmacy and Research Institute of Pharmaceutical Sciences, Gyeongsang National University, Jinju 52828, Republic of Korea

[f]KU-KIST Graduate School of Converging Science and Technology, Korea University, Seoul 02841, Republic of Korea

*Correspondence to: ysyang@kist.re.kr (Y. Yang), sunkim@kist.re.kr (S.H. Kim)

**Supporting Figures ------------------------ 2**

**Supporting Table --------------------------- 9**

# Supporting figures

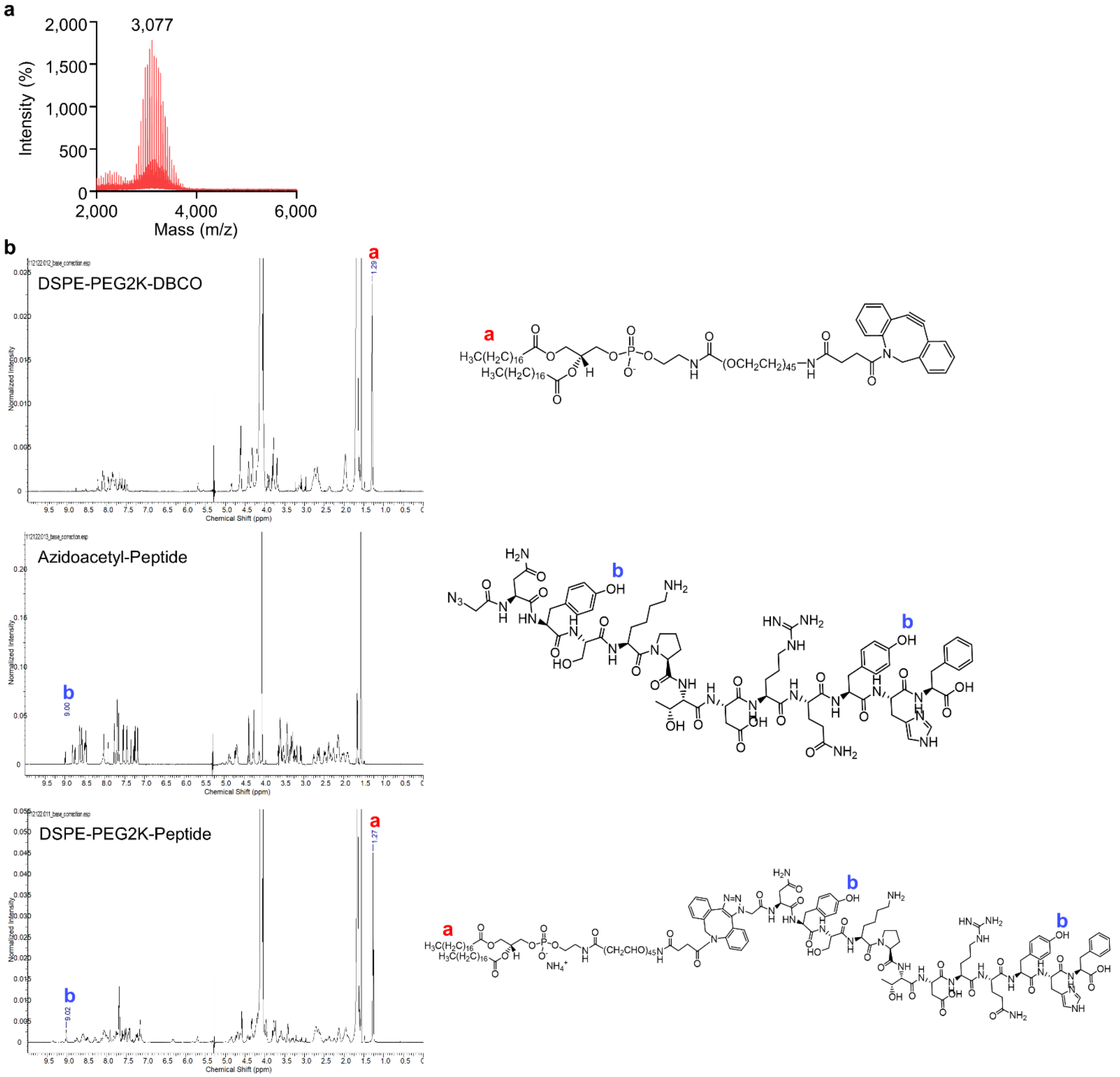


**Figure. S1: Mass and $^1$H NMR spectrum of components.**

**a** MALDI-TOFTOF mass spectrometric analysis of DSPE-PEG2K-DBCO using DHB matrix. **b** $^1$H NMR data and the structural formula of DSPE-PEG2K-DBCO, azidoacetyl PD-L1 binding peptide, and DSPE-PEG2K-Pep. Synthesis of DSPE-PEG2K-Pep was examined using $^1$H NMR; Both characteristic peaks, one at 9.0 ppm for the hydroxyl group in serine, and the other at 1.2 ppm for the hydrocarbons in the lipids, were confirmed.

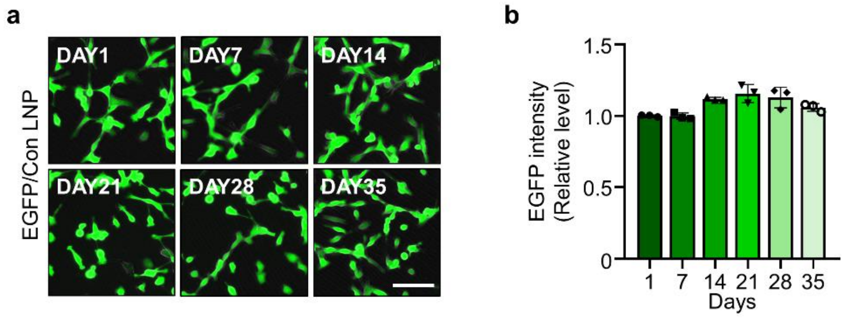


**Figure. S2: Stability of EGFP/Con LNPs.**

**a** Representative fluorescence images and **b** a quantified graph showing the transfection efficiency of EGFP/Con LNPs in CT26.CL25 cells at various time points after storage at 4 °C. Scale bar: 100 µm. Data presented as mean ± SD, $n = 3$.

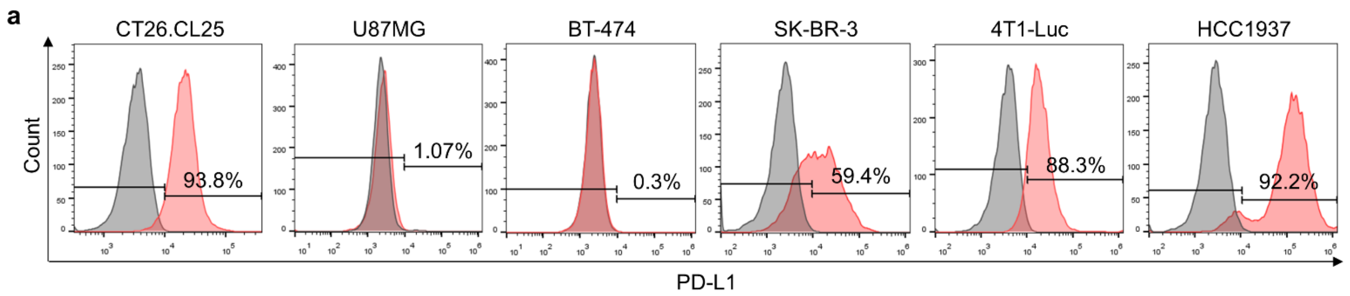


**Figure. S3: PD-L1 expression levels on different cancer cell surfaces.**
**a** Flow cytometry histograms showing PD-L1 expression on the surface of CT26.CL25, U87MG, BT-474, SK-BR-3, 4T1-Luc, and HCC1937 cells. The grey histogram represents the isotype control, while the red histogram represents the APC-conjugated PD-L1 antibody-treated group.

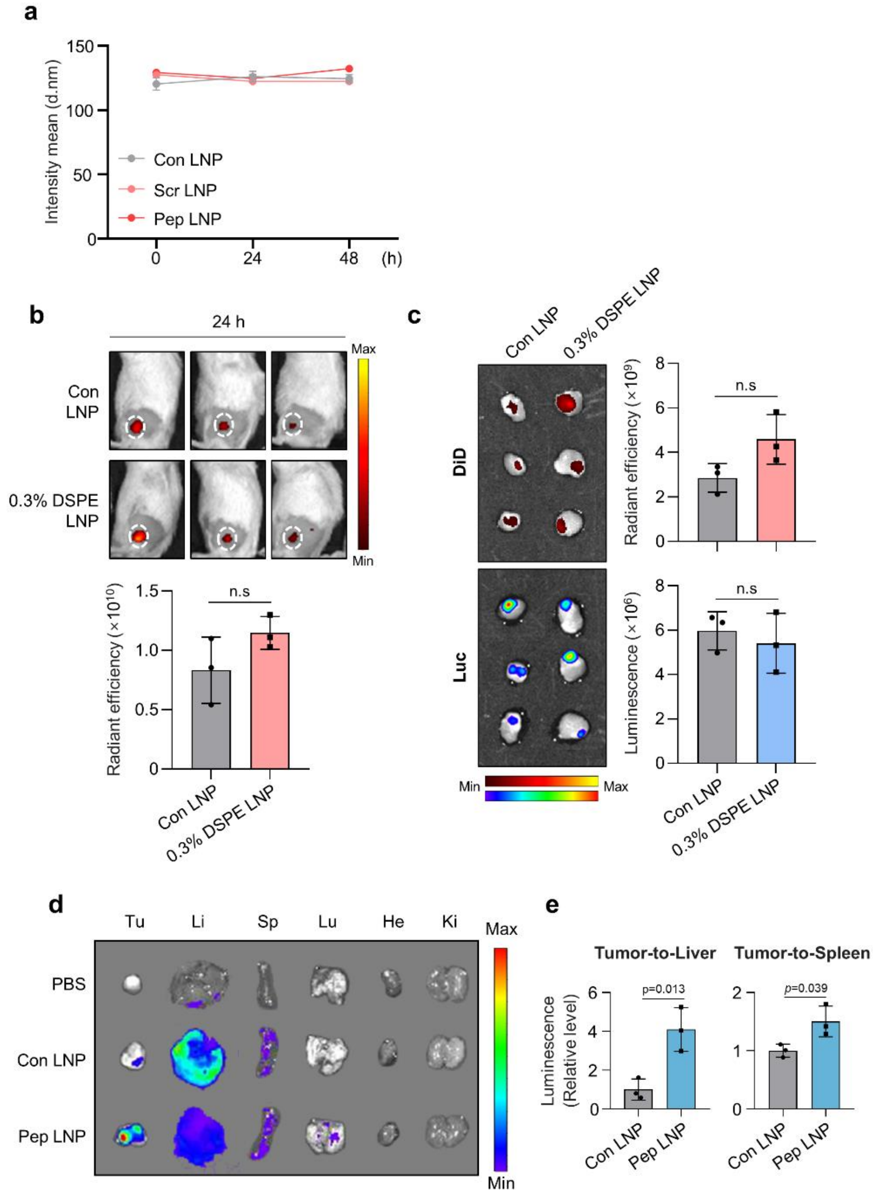


**Figure. S4: Stability and biodistribution of different LNPs.**

**a** Luc/Con, Scr, or Pep LNPs were incubated in 100% mouse serum at RT for different time points, and their size intensity mean was measured using DLS. **b** DiD fluorescence imaging and tumor localized fluorescence quantification of CT26.CL25 subcutaneous BALB/c mice model 24 h after intravenous injection of DiD-labeled Luc/Con LNPs or LNPs containing 0.3% DSPE-PEG2K (0.3% DSPE LNP). **c** Fluorescence and luminescence image with quantified graphs of tumors harvested at 24 h post-injection. **d** Luminescence imaging of harvested organs of 4T1-breaing BALB/c mice and **e** quantification of tumor luminescence level, with normalized to that of liver or spleen. All data is presented as mean ± SD, $n = 3$. Unpaired t-test.

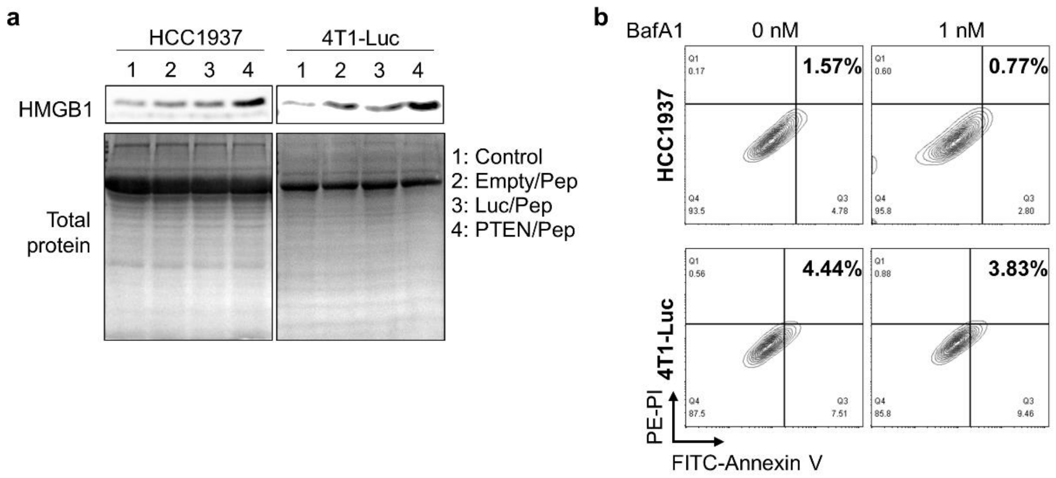


**Figure. S5: Western blot of HMGB1 and examination of apoptotic effect of BafA1.**
**a** Western blot image of HMGB1 and the Coomassie blue staining image of total protein. Cancer cells were treated with various LNPs, and after 48 h, the supernatant was collected to determine the HMGB1 protein levels. **b** The Annexin V/PI apoptosis assay demonstrates the absence of cellular toxicity at 1 nM BafA1 concentration.

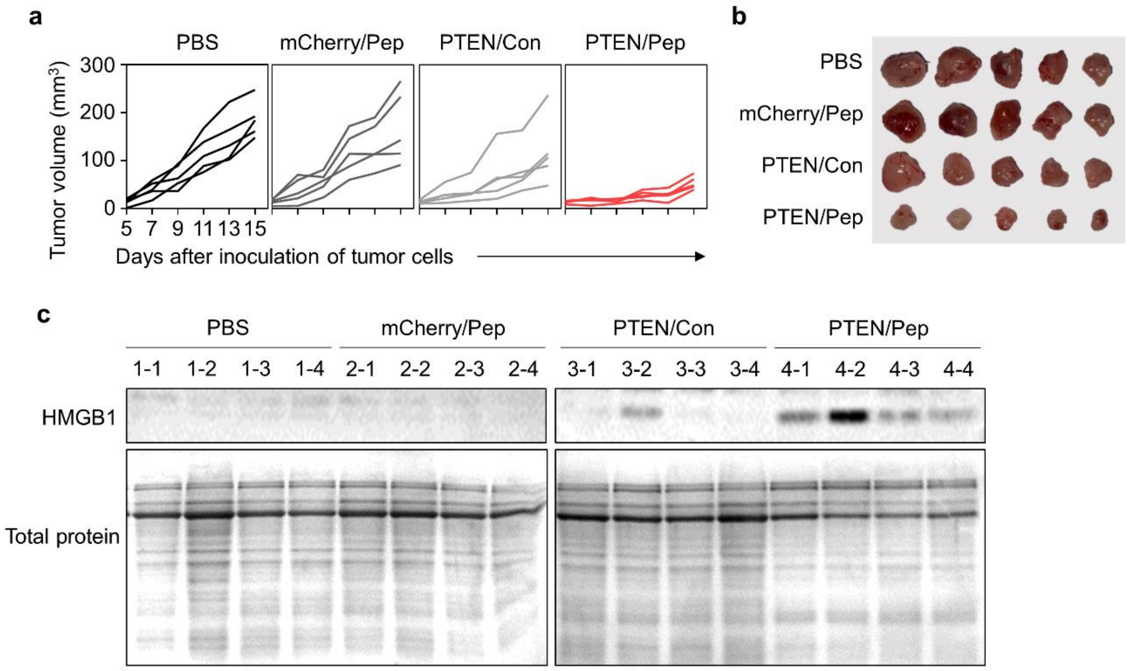


**Figure. S6: Tumor suppressive effect of PTEN/Pep LNPs.**
**a** Individual tumor growth curves. The size of the tumor was measured on days 5, 7, 9, 11, 13, and 15 post-inoculation of cancer cells. **b** Excised tumor images. **c** Western blot image of HMGB1 protein and Coomassie blue staining of total protein in the tumor supernatants.

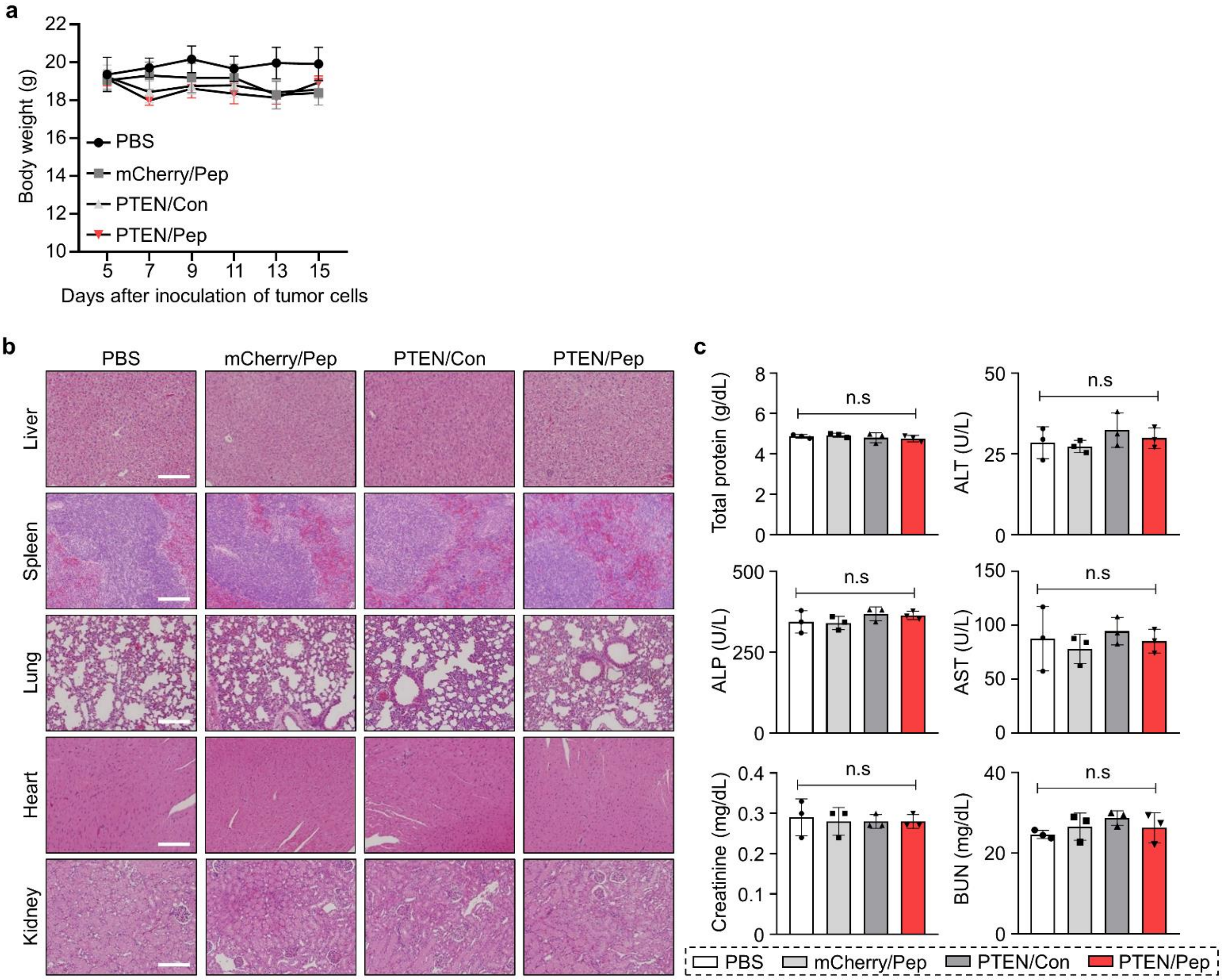


**Figure. S7: Safety profiles of different LNPs.**

**a** Mice's body weight. Mice were monitored during the experiment. **b** H&E staining images of the liver, spleen, lung, heart, and kidney tissues. The organs were collected 48 h post-injection with PBS, mCherry/Pep, PTEN/Con, and PTEN/Pep LNPs. Scale bar: 200 µm. **c** Blood chemistry analysis. The levels of total protein, ALT, AST, ALP, BUN, and creatinine in serum were measured. Data presented as mean ± SD, *n* = 3 (n.s, not significant; One-way ANOVA with Tukey's post-hoc test).

# Supporting Table

| | | | |
|---|---|---|---|
| 1 | pIVT-SDM/PTEN | For | 5’-GCTTGCCTGCAGGTCGACTCTAGGCCACCATGGATTACAAG-3’ |
| 2 | pIVT-SDM/PTEN | Rev | 5’-GGCGAATTCGAGCTCGGTACCCGGGCGAGTCAGACTTTTGTAA-3’ |
| 3 | PTEN template | For | 5’-CACTATAGAAGAGAAGGAATTAATACG-3’ |
| 4 | PTEN template | Rev | 5’-TTTTTTTTTTTTTTTTTTTTTTTTTTTTTTTTTTTTTTTTTTTTTTTTTTTTTT TTTTTTTTTTTTTTTTTTTTTTTTTTTTTTTTTTTTTTTTTTTTTTTTTTTTTTTT TTTTTTTTTTAGAATGT-3’ |

**Table. S1: List of primers**
